# Photothermal Amplification via Nanorobotic Swarming Dynamics

**DOI:** 10.1002/advs.76128

**Published:** 2026-06-15

**Authors:** Qinglong Wang, Lin Su, Zhengxin Yang, Yihang Jiang, Haojin Yang, Qianqian Wang, Li Zhang, Ben Wang, Jiajia Wang

**Affiliations:** ^1^ School of Public Health Guangzhou Medical University Guangzhou China; ^2^ Department of Mechanical and Automation Engineering The Chinese University of Hong Kong Hong Kong China; ^3^ Department of Industrial and Systems Engineering The Hong Kong Polytechnic University Hong Kong China; ^4^ The Suzhou Institute of Biomedical Engineering and Technology Chinese Academy of Sciences Suzhou China; ^5^ Jiangsu Key Laboratory for Design and Manufacturing of Precision Medicine Equipment School of Mechanical Engineering Southeast University Nanjing China; ^6^ Department of Robotics School of Mechanical Engineering Southeast University Nanjing China; ^7^ Chow Yuk Ho Technology Centre for Innovative Medicine The Chinese University of Hong Kong Hong Kong China; ^8^ Department of Surgery The Chinese University of Hong Kong Hong Kong China; ^9^ College of Chemistry and Environmental Engineering Shenzhen University Shenzhen China

**Keywords:** magnetic actuation, photothermal amplification, swarming dynamics, synergistic treatment

## Abstract

Photothermal therapy represents a promising therapeutic approach due to its non‐invasiveness and spatiotemporal controllability. However, conventional nanoparticle‐based systems are limited by low conversion efficiency, quick heat dissipation, and the high dosages required for sufficient therapeutic hyperthermia. Although micro/nanorobotic platforms improve targeting, they still face challenges in achieving adequate localized heat safely, often requiring high material concentrations that risk vascular complications. To address these limitations, this work introduces a strategy leveraging magnetically regulated swarming dynamics to amplify photothermal conversion. With designed magnetic actuation, building blocks are organized into the reconfigurable microswarm, achieving localized densification that minimizes heat dissipation and achieves photothermal amplification under near‐infrared (NIR) light. Compared with the dispersive state, the microswarm exhibits a 23 °C enhancement owing to its boosted photothermal conversion efficiency and great thermal stability across variable scales, effectively overcoming rapid heat dissipation in dynamic environments. Besides, magnetically controlled reconfiguration allows tunable heating areas, balancing spatial coverage and therapeutic intensity. Compared with dispersive systems, a 7‐fold improvement in the cancer cell‐killing efficiency of the microswarm can be achieved via photothermal amplification. This work establishes a photothermal amplification strategy to overcome limitations of passive diffusion and dosage dependence, pioneering a versatile nanorobotic platform for precision photothermal‐based treatment.

## Graphical abstract

This study explores a novel approach to enhance photothermal therapy using magnetically regulated microswarms. It details the development of magnetic nanoparticles organized into dynamic structures that improve heat retention and conversion efficiency. The research highlights the potential for tunable heating and synergistic treatment strategies, paving the way for advanced precision therapies in cancer treatment.

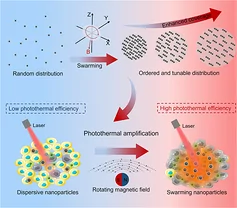

## 1 Introduction

Photothermal therapy has emerged as a promising technology for treating bacterial infection [1, 2, 3], neurodegenerative diseases [4, 5, 6], or cancer [7, 8, 9, 10, 11], etc. Its core mechanism lies in utilizing light sources with good tissue penetration to achieve localized hyperthermia (typically above 42°C) within the lesion area, thereby triggering responsive drug release or inducing apoptosis of diseased cells. As the thermal effect is strictly confined to the targeted area, damage to surrounding healthy tissues can be minimized [12, 13, 14, 15]. Despite its advantages of noninvasiveness and spatiotemporal controllability, traditional photothermal therapy relying on passive nanoparticle diffusion faces inherent limitations, including generally low photothermal conversion efficiency, rapid heat dissipation in biological environments, and the frequent requirement for high systemic dosages of nanomaterials to achieve sufficient hyperthermia, which significantly increases the risks of non‐specific uptake and systemic toxicity, thereby exacerbating off‐target effects. These challenges are further amplified in deep‐located lesions, where light penetration depth restricts effective energy delivery. Current strategies to enhance photothermal conversion, such as plasmonic nanostructure optimization [16, 17] and heterojunction design [18, 19], often neglect the fundamental mechanisms of heat dissipation in dynamic physiological environments, leaving the energy localization challenge unresolved.

Recent advances in micro/nanorobotics offer a new avenue to overcome these obstacles. By endowing nanoparticles with autonomous or externally driven motility and navigation capabilities, these systems achieve active targeting to the lesion region, thereby potentially reducing reliance on high systemic drug doses required in traditional approaches [20, 21, 22, 23, 24, 25, 26, 27, 28, 29, 30]. For instance, Mg‐based microrobots achieve high drug delivery efficiency in colon models through NIR‐triggered release mechanisms [31], while P_2_W_18_Fe_4_ polyoxometalate nanomotors demonstrate enhanced tumor penetration via self‐propulsion [32]. Yan et al. reported a PDA‐encapsulated nanomotor with fluorescent indocyanine green (ICG) immobilized on the PDA shell, which can generate favorable photothermal heat and emit fluorescence with NIR light [33]. Nevertheless, existing micro/nanorobotic systems still face bottlenecks in achieving efficient photothermal therapy: to reach the necessary therapeutic temperatures at the target site, high local material loading is often still required, which may pose risks of local vascular occlusion and inflammatory responses. Therefore, developing new strategies that can enhance localized energy conversion while ensuring biosafety has become crucial for advancing photothermal‐based therapy from the laboratory toward clinical application.

**FIGURE 1:**
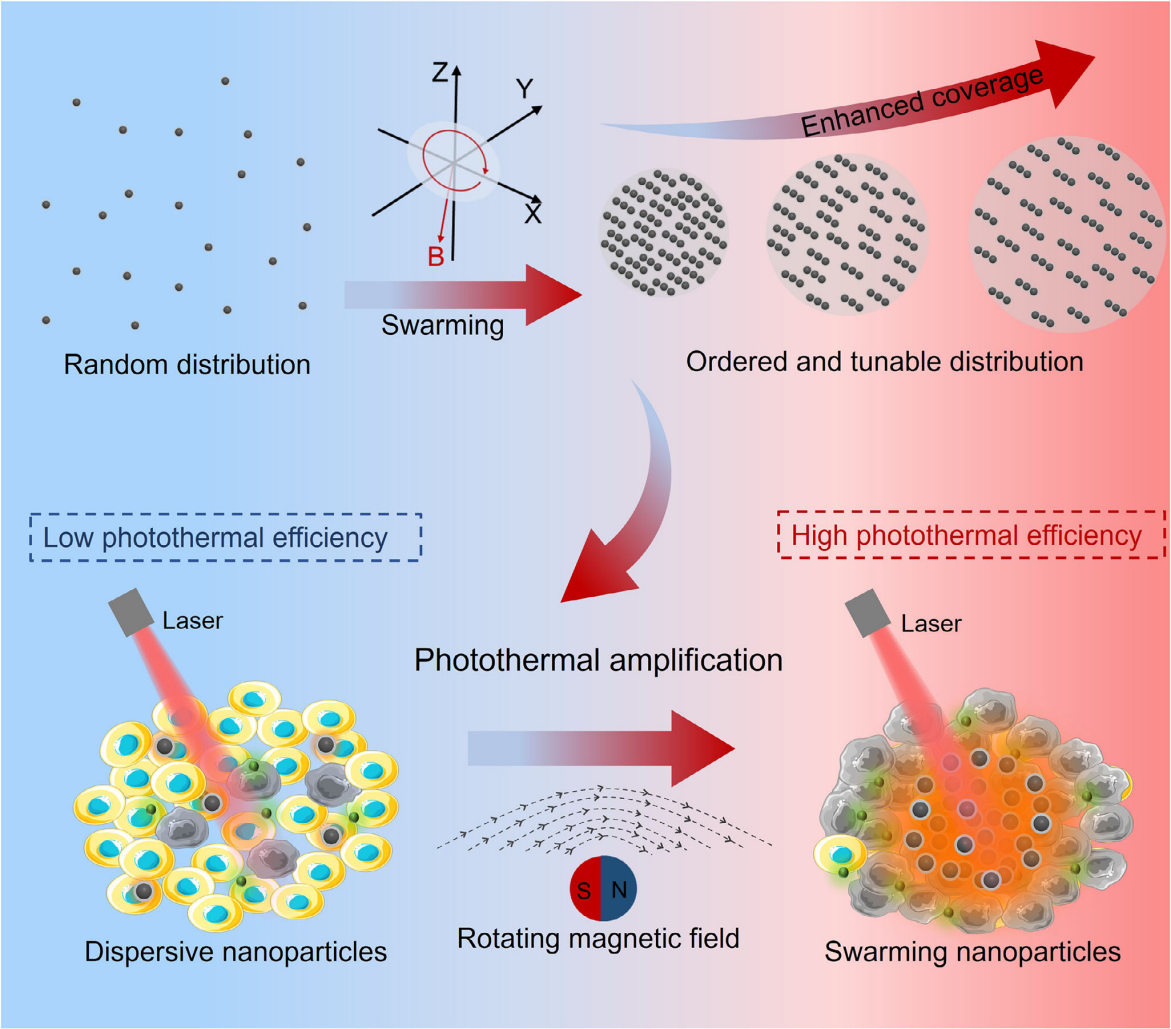
The schematic shows the enhanced photothermal conversion via the swarming dynamics of the magnetic nanoparticles.

This work addresses these limitations through magnetically programmed emergent swarming dynamics, which leverage collective behavior to amplify photothermal efficacy without relying on complex nanostructural design (Figure 1). Fe_3_O_4_@PDA@‐DOX nanoparticles are employed as building blocks to form reconfigurable microswarms under rotating magnetic fields, boosting photothermal conversion through synergistic spatial densification and thermal localization. Localized nanoparticle densification via swarming control increases the areal nanoparticle density from 0.127 to 7.53 µg/mm^2^ and effectively suppresses thermal dissipation, enabling a 23°C temperature elevation. Furthermore, dynamic morphology adaptation permits tunable heating areas while maintaining stable hyperthermia across various conditions under NIR light. Besides, NIR‐accelerated release of DOX synergized with photothermal ablation, yielding a 7‐fold improvement in cancer cell lethality compared with dispersive nanoparticles. To improve the delivery efficiency, ultrasound (US) imaging‐guided tracking and navigation ensure an access rate of over 80% against the blood flow of 4.34 cm/s, overcoming challenges associated with hemodynamic clearance. In this system, the individual magnetic nanoparticles serve as the building blocks. Under the precise modulation of a programmed rotating magnetic field, these passive building blocks dynamically assemble into a highly organized microswarm, acting as the robotic entity. Through emergent swarming dynamics, this robotic entity acquires capabilities including controllable navigation in dynamic environments, on‐demand morphological reconfiguration, and the ability to amplify localized photothermal conversion. The proposed strategy transcends the conventional efficacy‐biosafety trade‐off by elevating energy conversion via emergent swarming dynamics, potentially facilitating the development of nanorobotic‐based therapeutic platforms for efficient and localized hyperthermia therapies.

## 2 Results and Discussion

### 2.1 Material Characterizations

**FIGURE 2:**
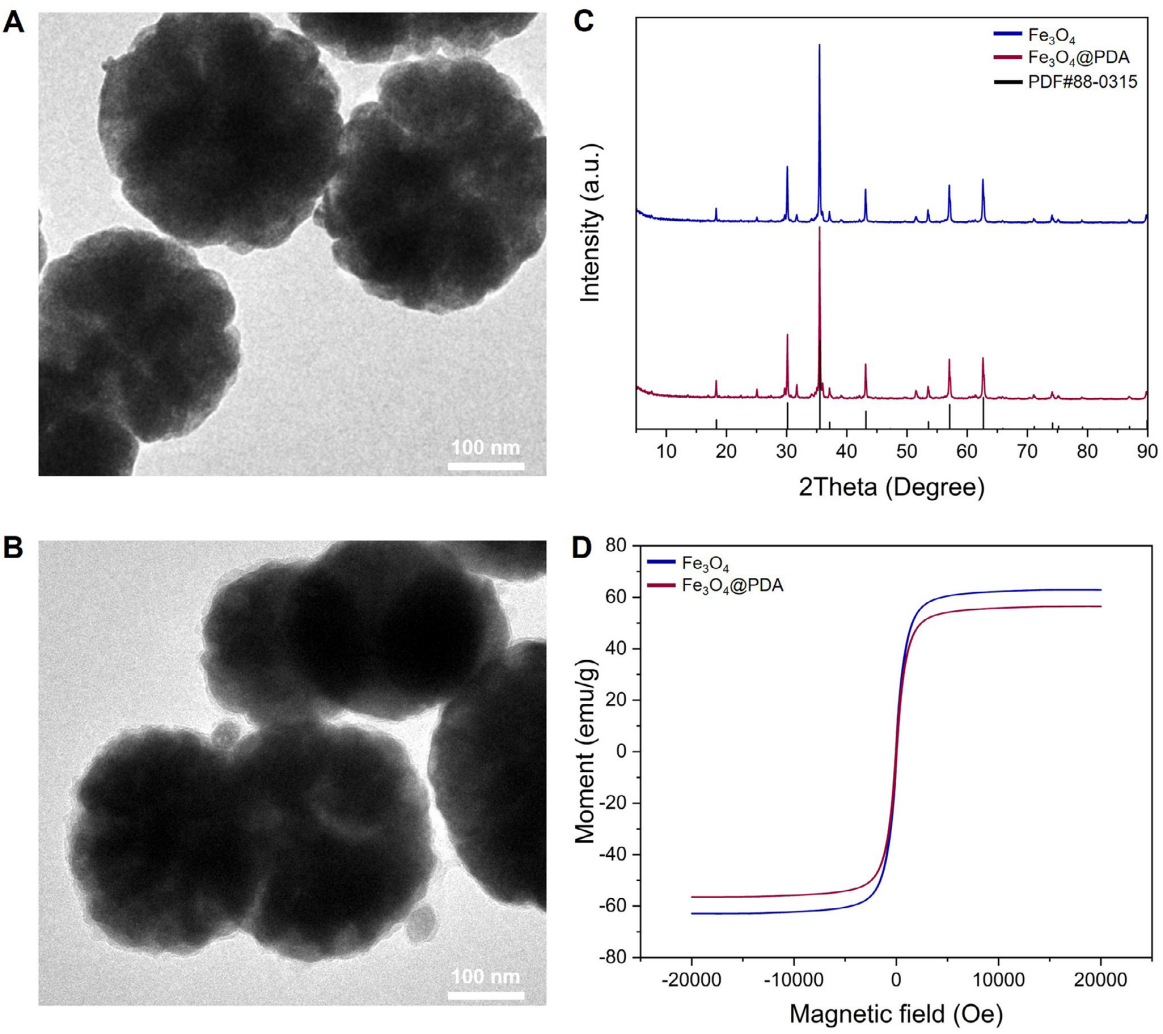
Material characterization of the prepared magnetic nanoparticles. (A) Transmission electron microscope (TEM) imaging of Fe_3_O_4_ nanoparticles and (B) Fe_3_O_4_@PDA nanoparticles. (C, D) X‐ray diffraction (XRD) patterns and magnetic hysteresis loops of Fe_3_O_4_ and Fe_3_O_4_@PDA nanoparticles.

The Fe_3_O_4_ nanoparticles were synthesized by the thermal‐solvent method [34]. The diameter of the nanoparticles was relatively uniform in the range of 200–300 nm (Figure 2A). Then, a thin PDA coating was introduced through a polymerization process to further improve the photothermal effect, as evidenced by the TEM image in Figure 2B. Moreover, XRD analysis was conducted to confirm the components of the prepared nanoparticles. XRD patterns of pure Fe_3_O_4_ and Fe_3_O_4_@PDA nanoparticles matched well with the standard JCPDS card (88–0315). The attenuated peak intensities in Fe_3_O_4_@PDA pattern indicated reduced crystallinity after successful PDA coating (Figure 2C). Surface charge characterization was conducted to further validate the PDA modification (Figure S1). The zeta potential of bare Fe_3_O_4_ nanoparticles peaked at −23.13 ± 0.21 mV, demonstrating a characteristic negative surface charge. After PDA coating, the zeta potential significantly shifted to −3.37 ± 1.58 mVbecause of the protonation behavior of abundant amine groups. Vibrating sample magnetometry (VSM) demonstrated preserved ferrimagnetism in both samples, with saturation magnetization values decreasing from 62.8 emu/g (Fe_3_O_4_) to 56.4 emu/g (Fe_3_O_4_@PDA) (Figure 2D). This 10.2% reduction in magnetic moment was primarily attributed to the mass contribution of the diamagnetic PDA layer. Importantly, both the Fe_3_O_4_ and Fe_3_O_4_@PDA nanoparticles retained high saturation magnetization, which is crucial for generating a sufficient magnetic response to enable subsequent swarm generation and locomotion under applied magnetic fields.

### 2.2 Photothermal Amplification via Swarming Dynamics

**FIGURE 3:**
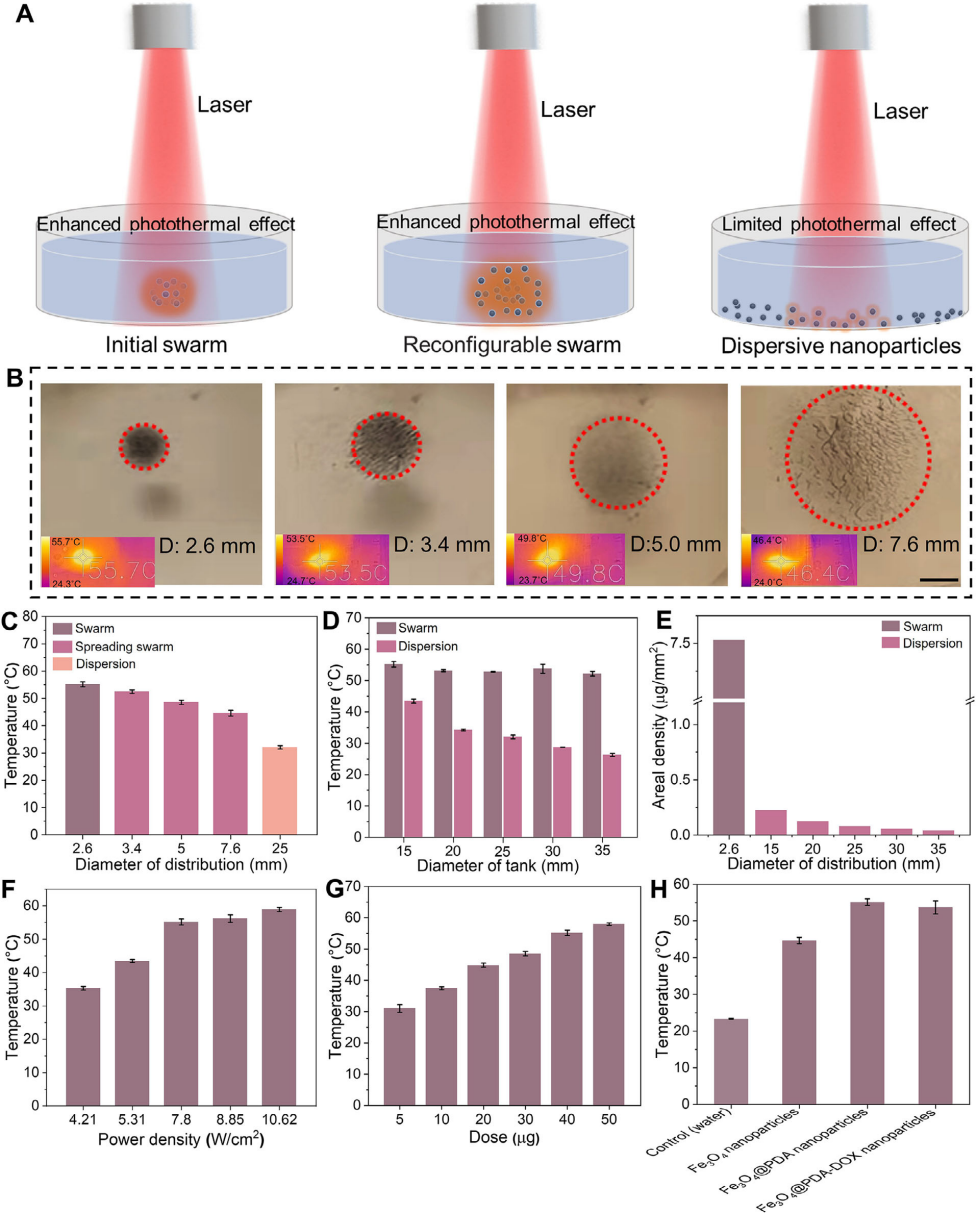
Photothermal amplification via magnetic‐controlled swarming dynamics. (A) Schematic of the photothermal effect of the initial swarm, reconfigurable swarm, and dispersed nanoparticles. (B) Photographs of the swarm with different distribution densities controlled by the magnetic field, and the inset images show the final temperature of each state. Dose: 40 µg, magnetic field strength: 34.2 mT, input frequency: 3 Hz, laser power density: 7.8 W/cm^2^. Scale bar: 2 mm. (C) The temperature change of dispersion, swarm, and spreading swarm in the tank with a diameter of 25 mm. Dose: 40 µg, magnetic field strength: 34.2 mT, input frequency: 3 Hz, laser power density: 7.8 W/cm^2^. (D) The temperature comparison of the swarm and dispersion in tanks with different diameters. Dose: 40 µg, magnetic field strength: 34.2 mT, input frequency: 3 Hz, laser power density: 7.8 W/cm^2^. (E‐G) The influence of laser power density and swarm dose on the photothermal effect. Magnetic field strength: 34.2 mT, input frequency: 3 Hz, tank diameter: 25 mm. (H) The temperatures of the swarm consisting of Fe_3_O_4_, Fe_3_O_4_@PDA, and Fe_3_O_4_@PDA‐DOX nanoparticles, with pure water used as a control group. Dose: 40 µg, magnetic field strength: 34.2 mT, input frequency: 3 Hz, laser power density: 7.8 W/cm^2^, tank diameter: 25 mm. The error bars represent the standard deviation (SD) of three experiments.

The photothermal performance of magnetically reconfigured nanoparticle swarms was quantitatively evaluated under NIR light. As illustrated in Figure 3A, precise magnetic actuation enabled dynamic control over the microswarm morphology [35, 36, 37]. The diameter of the initial swarm was 2.6 mm. By modulating the parameters of the rotating magnetic field, the swarm could be dynamically reconfigured, expanding its diameter to 3.4, 5.0, and 7.6 mm, corresponding to different spatial configurations (Figure 3B). Such densification induced by spatial reorganization led to the initial swarm with a high areal density of 7.53 µg/mm^2^ (Figure S2), generating localized hyperthermia at 55.2°C (Figure 3C). This represents a 59‐fold enhancement in areal density and around 23°C increase in temperature compared to dispersed nanoparticles (Figure S3), demonstrating the pivotal role of the swarming behavior in suppressing thermal dissipation. Although the maximum thermal output occurred in high‐density configurations, spatial coverage remained constrained. To address this concern, a designed conical magnetic field was applied to achieve an 8.5‐fold improvement in the spatial coverage area from 5.31 to 45.36 mm^2^, while maintaining sufficient therapeutic hyperthermia at 44.6°C (Figure 3C, see also Video S1). This inverse correlation between areal density and spatial coverage (Video S2) established a tunable energy delivery approach, offering a means to precisely balance the trade‐off between thermal intensity and treatment volume. Taken together, such on‐demand reconfigurability enables adaptive photothermal therapy optimized for heterogeneous lesion geometries. The environmental adaptability of the microswarm was further evaluated under spatially confined conditions using spherical tanks with various diameters. As quantified in Figure 3D, microswarms maintained stable hyperthermia (around 53.0°C) across all confinement scales, demonstrating excellent thermal stability.

This photothermal stability across scales highlights a key advantage of the swarming strategy: overcoming the rapid heat dissipation that affects dispersed systems with low density in unconfined or flowing environments. By comparison, dispersed nanoparticles with random distribution suffered a dramatic decline in efficacy, as the temperature decreased from 43.4°C to 26.3°C with tank diameter increasing from 15 to 35 mm, which can be explained by rapid heat dissipation and reduced areal density. This difference originated from swarm‐induced volumetric heat accumulation, whereby magnetic field‐guided densification achieved a 132‐fold areal density enhancement compared with the dispersive state in the 30 mm tank (Figure 3E), effectively creating localized thermal conversion that resists environmental heat dissipation. As a result, such a microswarm‐based particle redistribution strategy offers an alternative strategy to overcome photothermal instability in dynamic and complex physiological environments.

The photothermal response of magnetically assembled microswarms can be tuned through parameter optimization either by laser power or nanoparticle dosage. As demonstrated in Figure 3F, the temperature exhibited a linear relationship to laser intensity, achieving precise thermal control from 35.3°C to 58.9°C, spanning subtherapeutic to tumor‐ablative regimes. Besides, spatial power modulation can be achieved through real‐time laser tip positioning (Figure S4), enabling dynamic thermal control. Dosage‐dependent photothermal regulation revealed a critical threshold: 20 µg of the microswarm under 5.31 W/cm^2^ laser irradiation was sufficient to exceed the 42°C hyperthermia threshold (Figure 3G), representing a 5‐fold reduction in dosage compared to the nanoparticle dispersion (100 µg) with a similar thermal effect (41.5°C). PDA functionalization further enhanced photothermal conversion, yielding a 10.5°C temperature elevation versus bare Fe_3_O_4_ nanoparticles (Figure 3H). Notably, DOX loading into the PDA matrix preserved comparable energy conversion capability, enabling synergistic therapy without photothermal compromise.

**FIGURE 4:**
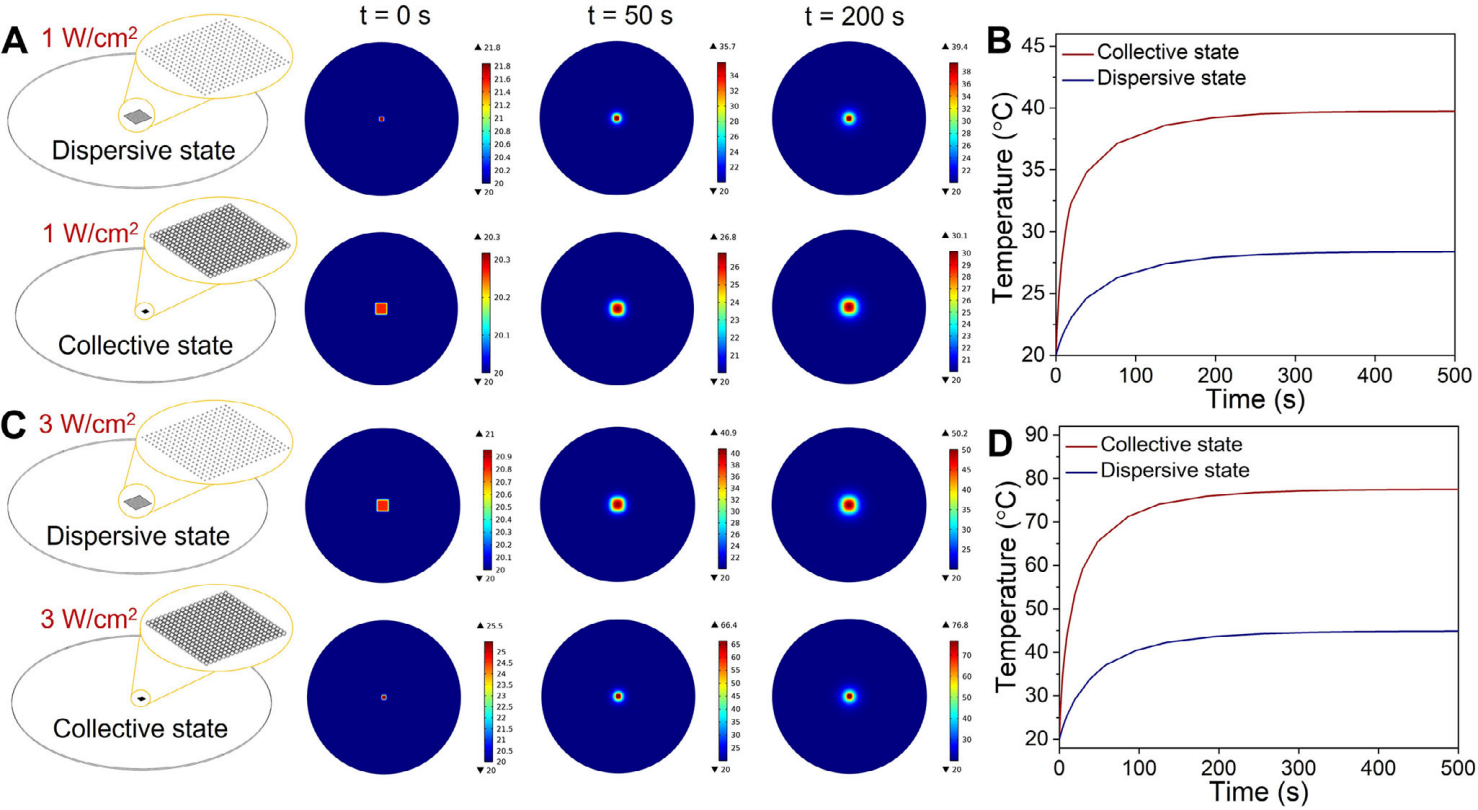
The simulation of the photothermal effect of nanoparticles in collective and dispersive states. (A) The simulation results of the photothermal effect of nanoparticles in collective and dispersive states with a laser power density of 1 W/cm^2^. (B) Temperature change of nanoparticles in collective and dispersive states with a laser power density of 1 W/cm^2^ over time. (C) The simulation results of the photothermal effect of nanoparticles in collective and dispersive states with a laser power density of 3 W/cm^2^. (D) Temperature change of nanoparticles in collective and dispersive states with a laser power density of 3 W/cm^2^ over time.

The photothermal performance of magnetic nanoparticles in swarming and dispersive states under varying laser power densities (1 and 3 W/cm^2^) was compared via simulations. The thermal maps (Figure 4A,B, see also Video S3) demonstrate a pronounced contrast in heat generation efficiency between collective and dispersive states. At t = 50 s, the collective state exhibits rapid temperature escalation to 35.5°C, while the dispersive state remains below 26°C, suggesting superior light‐to‐heat conversion efficiency in the collective state. By t = 200 s, the collective assembly achieves thermal equilibrium at approximately 39°C, contrasted with the dispersive system gradually ascending to 27°C. As laser power density increased to 3 W/cm^2^ (Figure 4C,D), both systems exhibited enhanced thermal responses but maintained similar state‐dependent behaviors. More than a 30°C difference can be observed between those two states after thermal equilibrium. This observation aligns well with our experimental results, confirming that swarming dynamics can dramatically amplify the macroscopic photothermal effect. These findings provide critical insights for designing nanorobotic systems optimized for photothermal‐based applications, balancing the trade‐off between maximizing localized heat intensity and managing the spatial coverage of hyperthermia.

To elucidate the physical mechanism underlying the observed scale‐invariant thermal stability, we introduce a heat transfer model based on Newton's law of cooling. At thermal equilibrium, the photothermal heat generation rate (*Q_gen_*) equals the heat dissipation rate (*Q_diss_*) to the surrounding environment (*Q_gen_* = *Q_diss_* ). According to Newton's law of cooling [38], the heat dissipation rate is generally expressed as *Q_diss_* =  *h* · *A* · Δ*T*, where *h* is the heat transfer coefficient, *A* is the effective surface area for heat exchange, and Δ*T* is the temperature difference between the heat source and the environment. The difference in thermal behavior between the dispersed and swarming states stems from their heat dissipation boundaries (*A*). For the dispersed state, the nanoparticles are randomly distributed, and heat generation is spatially dispersed across the entire liquid volume. Consequently, heat dissipation occurs at the macroscopic boundary of the container. As the container size increases, *A* increases significantly, and the system is forced to reach equilibrium at a drastically lower Δ*T*, leading to a rapid decline in the maximum temperature. Conversely, for the microswarm, the densely distributed nanoparticles act as a highly concentrated, localized heat source. The heat dissipation occurs directly at the boundary of the microswarm itself (*A_swarm_*). Therefore, the local boundary condition (*A_swarm_*) is determined solely by the microswarm's morphology. This robust thermal confinement ensures that the heat dissipation rate of the microswarm is relatively low, enabling the system to maintain a highly stable equilibrium temperature regardless of varying spatial confinements.

To quantitatively distinguish the photothermal enhancement arising from intrinsic material properties versus spatial densification, the photothermal conversion efficiency (PCE, η) was calculated using the standard Roper method based on the cooling curves (Figure S5). The results revealed that the surface polymerization of PDA significantly improved the near‐infrared absorption, increasing the intrinsic PCE of the swarm from 15.30% (bare Fe_3_O_4_) to 29.04% (Fe_3_O_4_@PDA) (Figure S5F). While the dispersed Fe_3_O_4_@PDA nanoparticles exhibited a low apparent PCE of only 5.41% (*T_max_* = 33.9°C) due to rapid heat dissipation into the bulk fluid, actuating them into the swarming state dramatically elevated the apparent PCE to 29.04% (*T_max_* = 47.0°C) (Figure S5E,F). Since the intrinsic light‐to‐heat conversion capability of individual nanoparticles remains constant, this 5.3‐fold improvement in the apparent PCE is directly attributed to the robust thermal confinement induced by the swarming behavior. The spatial densification drastically reduces the effective heat dissipation boundary, preventing rapid energy loss and allowing the generated heat to be highly concentrated for localized hyperthermia.

### 2.3 Controllable Locomotion of the Microswarm

**FIGURE 5:**
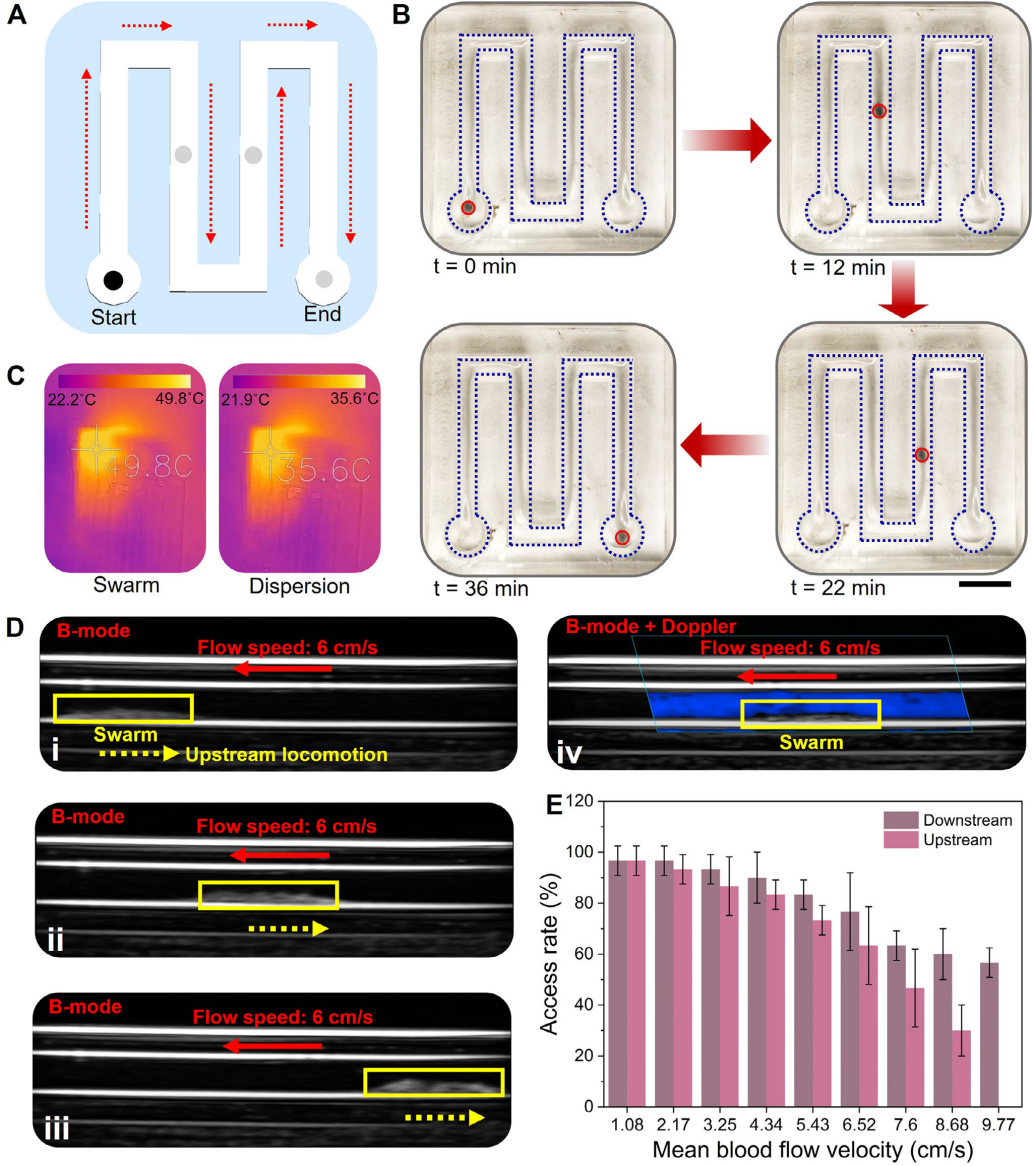
Controllable locomotion of the microswarm in the channel and vessel. (A) Schematic of microswarm's controllable locomotion in a model with a tortuous channel. (B) Representative video frames of the microswarm locomotion process at different time intervals. Scale bar: 5 mm. (C) Comparison of the photothermal effect of nanoparticles in collective and dispersive states after arriving at the target site. (D) Ultrasound (US) imaging of the microswarm in the silicone tube with an inner diameter of 2 mm, filled with whole pig blood. (i–iii) The typical video frames of the swarm's upstream locomotion in the flowing blood (mean velocity of 6 cm/s). (iv) US imaging of the swarm with B‐mode and Doppler signals in the flowing blood (mean velocity of 6 cm/s). (E) The access rate of the microswarm in the whole blood with various mean flow velocities. Magnetic field strength: 62.7 mT, input frequency: 3 Hz, navigation distance: 5 cm, dosage: 100 µg. The error bars represent the SD of three experiments.

The schematic illustrates the ability of the designed microswarm to maintain structural integrity while smoothly traversing tortuous channels (Figure 5A). Under the rotating magnetic field, individual nanoparticles were dynamically attracted to form magnetic chains via the dipole‐dipole interactions. These chains then further dynamically coalesced into a coherent microswarm, driven by the combined effects of magnetic interactions and hydrodynamic flows [39, 40]. After microswarm formation, controllable locomotion was realized by magnetic actuation with a locomotion velocity of 40 µm/s (Video S4). The microswarm was able to continuously move for a relatively long distance, and 91.7% of the nanoparticles successfully arrived at the target region after 36 min. This precise navigation capability stems from dynamic collective behavior under rotating magnetic fields, enabling on‐demand shape transformation to conform to confined geometries without structural disintegration, which is crucial for traversing tortuous paths. After that, the photothermal effect of the microswarm and dispersed nanoparticles in the targeted region was investigated (Figure 5C). Compared with dispersion, about a 14°C temperature rise was observed for the microswarm as shown in Figure 5B, which further confirmed the enhanced photothermal conversion via swarming regulation. Real‐time imaging and tracking of the microswarm permit precise delivery of therapeutic agents in complex and dynamic environments. Here, the ultrasound (US) modality was employed to track and navigate the swarm in the vessel with flowing blood (Video S5). As observed in Figure 5D (i–iii), the swarm showed clear imaging signals (B‐mode) in the tube (inner diameter of 2 mm) filled with whole blood. The microswarm could move against the blood flow (mean blood flow velocity of 6 cm/s) while maintaining its structural integrity. Moreover, obvious Doppler signals induced by the dynamic bloodstream were observed in Figure 5D (iv), while the microswarm profile can also be recognized by the signal differences from the surrounding environment, indicating that precise tracking of the microswarm can be realized with multimodal US imaging. Furthermore, to verify the ability of the microswarm resisting the bloodstream, the access rates were quantitatively analyzed. In flowing blood, the microswarm exhibited robust magnetically controlled movability both in the upstream and downstream. For downstream locomotion (where magnetic propulsion aligns with flow direction), the microswarm arrived at the targeted site with an access rate over 83% when the blood flow was lower than 5.77 cm/s (Figure 5E). By comparison, the access rate was greatly reduced in upstream blood (motion against the flow), reaching 0% with a mean blood flow velocity of 9.77 cm/s. The microswarm's superior magnetic responsiveness and precise near‐wall navigation provided excellent hemodynamic resistance against blood flow, offering opportunities for precise cardiovascular therapy.

### 2.4 In Vitro Cancer Cell Killing Test

**FIGURE 6:**
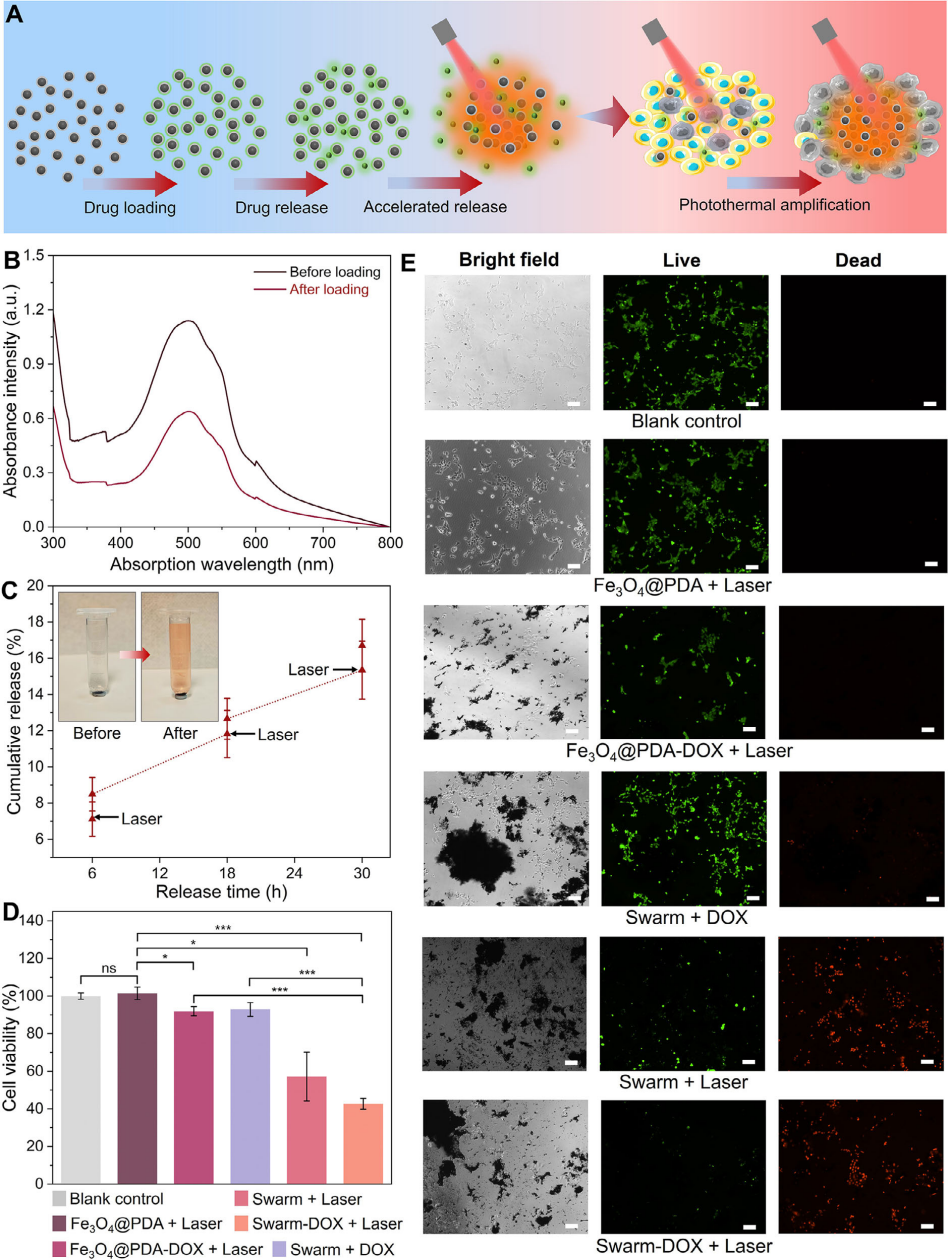
In vitro cancer cell killing test. (A) The schematic shows the drug loading, drug release, and photothermal amplification for cancer cell killing. (B) UV–vis absorbance spectra of DOX before and after loading. (C) Cumulative release percentage of DOX released from Fe_3_O_4_@PDA‐DOX nanoparticles (1 mg) over time, and the samples were treated with laser irradiation at different time points. Inset photos showing the color change before and after drug release. The error bars represent the SD of three experiments. (D) Cancer cell viability of various experimental groups in vitro. The error bars represent the SD of three experiments. The data were evaluated by Student's *t*‐test: ns, not significant; P values between 0.001–0.01 are given one asterisk; P values less than 0.001 are given two asterisks; and P values less than 0.0001 are given three asterisks. (E) Live/dead staining results of cancer cells with different treatments. The green and red fluorescence denote the live and dead cancer cells, respectively. Scale bar: 100 µm.

The Fe_3_O_4_@PDA nanoparticles were designed to serve dual functions as both photothermal generators and drug carriers. To characterize their drug delivery performance, we investigated drug loading capability and thermal‐induced accelerated release profile, as illustrated in Figure 6A. UV–vis spectra revealed a characteristic peak at 480 nm upon DOX loading and release (Figure 6B). The prepared sample achieved a high drug loading capacity of 315.7 µg/mg, and the drug concentration was calibrated using a standard absorption curve (Figure S6). As illustrated in Figure S7, UV–vis spectra showed the drug release profile at various time intervals (6, 18, and 30 h), and the absorbance intensity gradually increased from 0.20 to 0.41, with an accelerated release triggered by laser irradiation. The cumulative amount of released DOX was further analyzed as shown in Figure 6C. At 6 h, around 7.12% of DOX was released, and the value increased to 8.50% after laser irradiation, showing photothermally‐enhanced drug release. After 30 h, the percentage of released DOX increased to 16.7%, accounting for a weight of 52.7 µg. Moreover, the color change further verified the successful drug release from the prepared sample. As a result, Fe_3_O_4_@PDA nanoparticles demonstrated high drug‐loading capability, and laser irradiation enabled accelerated drug release, facilitating synergistic therapy through the chemo‐photothermal treatment. The heat not only directly ablates cells but also triggers on‐demand drug release, creating a combined therapeutic effect.

The magnetically controlled collective behavior of the microswarm demonstrated remarkable photothermal amplification through precise spatial densification. As a proof‐of‐concept, enhanced chemo‐photothermal for cancer cell killing was evaluated. In vitro evaluations revealed that dispersed Fe_3_O_4_@PDA nanoparticles exhibited limited therapeutic efficacy, maintaining nearly 100% cell viability after laser irradiation due to poor light‐harvesting capacity. In contrast, the configurable microswarm achieved 57.2% viability through swarming‐induced photothermal amplification (Figure 6D). Although chemo‐photothermal synergy could strengthen therapeutic outcomes, dispersed Fe_3_O_4_@PDA‐DOX nanoparticles still showed a limited cell‐killing effect. The cell viability reached 91.9% after treatment, mainly owing to the low amount of DOX released from the dispersed nanoparticles under laser irradiation. By comparison, swarming nanoparticles achieved targeted drug accumulation combined with hyperthermia‐triggered apoptosis, leading to a 7‐fold improvement in cell‐killing efficiency. The superior performance arises from the dual amplification: magnetically guided spatial focusing of both the photothermal agents and the chemotherapeutic drug to the target cells, coupled with hyperthermia‐triggered apoptosis and likely enhanced drug release/uptake. Live/dead staining analysis confirmed the superior therapeutic efficiency of swarm‐mediated photothermal treatment. As illustrated in Figure 6E, cell growth and proliferation were influenced negligibly by dispersive nanoparticles. In contrast, the majority of the cancer cells were killed by the microswarm due to the magnetically controlled localized hyperthermia. Taken together, the magnetically regulated swarming dynamics significantly boosted energy delivery efficiency, enabling spatiotemporally controlled photothermal therapy and transcending limitations of traditional nanoparticle‐based systems.

To further elucidate the biological mechanism underlying the enhanced chemo‐photothermal therapy, we performed flow cytometry to quantitatively analyze the cell death pathways (Figure S8). Consistent with the viability assays, the control and dispersed nanoparticle groups exhibited low apoptotic rates. In contrast, the combined synergistic treatment mediated by the microswarm (Swarm‐DOX + Laser) induced severe cellular apoptosis. The flow cytometry analysis revealed a remarkable total apoptosis rate of 42.88%, comprising 25.87% early apoptotic cells and 17.01% late apoptotic/necrotic cells. This comprehensive cellular evaluation provides solid biological evidence that the swarming‐induced robust thermal confinement not only facilitates direct thermal ablation but also accelerates localized DOX release, thereby synergistically activating apoptotic pathways to maximize the cancer cell‐killing efficacy.

### 2.5 In Vivo Locomotion and Photothermal Amplification of the Microswarm

**FIGURE 7:**
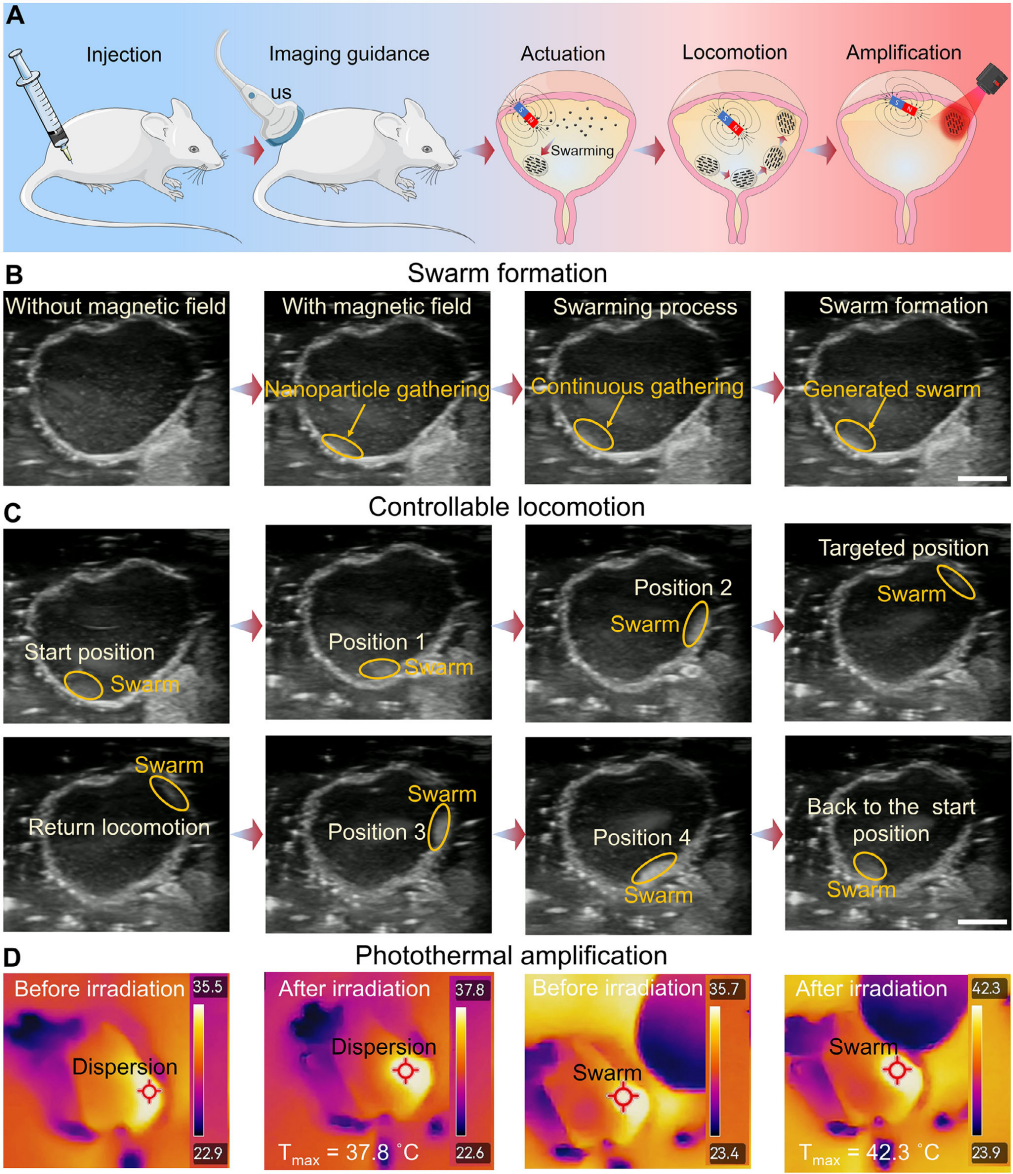
In vivo ultrasound‐guided locomotion and photothermal amplification of the microswarm. (A) Schematic illustration of the in vivo experimental procedure, including intravesical injection, ultrasound (US) imaging guidance, magnetic actuation for microswarm formation, controllable locomotion, and photothermal amplification within the bladder. (B) Real‐time ultrasound images showing the dynamic formation process of the microswarm under an applied magnetic field (Video S6). (C) Ultrasound images demonstrating the controllable and reversible locomotion of the microswarm along the bladder wall to a targeted position and back to the start position. (D) In vivo infrared thermal images and corresponding maximum temperatures (*T_max_*) of the nanoparticle dispersion and the microswarm before and after 808 nm laser irradiation. Scale bar: 5 mm.

To evaluate the structural stability, navigation ability, and therapeutic relevance of the microswarm in a complex physiological environment, we conducted in vivo experiments using a rat bladder model (Figure 7A). Following intravesical injection, real‐time ultrasound imaging demonstrated that the randomly dispersed nanoparticles rapidly assembled into a highly dense and structurally stable microswarm under a rotating magnetic field, successfully overcoming physiological fluid resistance (Figure 7B). Furthermore, the formed microswarm exhibited precise navigation capabilities in vivo. As shown in Figure 7C, by manipulating the external magnetic field, the microswarm was precisely steered from its start position, moving continuously along the curved and irregular inner wall of the bladder (via intermediate Positions 1 and 2), until it successfully reached the targeted region. Remarkably, the swarm also demonstrated highly controllable reversible actuation, successfully performing a return locomotion (via Positions 3 and 4) to navigate back to the initial start position. Throughout this entire navigation process along the tissue interface, the microswarm maintained its dense structure without dispersing into the surrounding physiological fluid, verifying its robust structural integrity and maneuverability. More importantly, we validated the scale‐invariant thermal stability of the microswarm in this large in vivo heat sink. Under 808 nm laser irradiation, the temperature of the dispersed nanoparticles only reached a maximum of 37.8°C (Figure 7D). Based on Newton's law of cooling (*Q_diss_* =  *h* · *A* · Δ*T*), this insufficient heating is attributed to the massive heat dissipation boundary (*A*) of the dispersed state, which causes the generated heat to rapidly dissipate into the surrounding large tissue volume. In contrast, when actuated into a microswarm, the heat dissipation boundary is drastically reduced to the localized surface of the swarm itself (*A_swarm_*). This robust thermal confinement effectively minimizes heat loss to the physiological environment, allowing the local temperature to rapidly elevate to 42.3°C (Figure 7D). This highly localized and amplified heating effect is sufficient to trigger effective photothermal therapy while sparing surrounding healthy tissues, thereby demonstrating the microswarm as a viable biomedical treatment strategy.

## 3 Conclusion

This study demonstrates a promising transition in photothermal therapy by exploiting emergent swarming dynamics to address the limitations of conventional nanoparticle‐based strategies. Unlike traditional approaches that rely on passive diffusion and high‐dose administration to compensate for inefficient photothermal conversion, the collective behavior of the microswarm enables a 23°C temperature elevation achieved through localized densification under the rotating magnetic field. The microswarm's reconfigurable morphology allows spatiotemporal control over thermal output and environmental adaptability, while maintaining stable hyperthermia (around 53°C) in varying environments where dispersed nanoparticles fail. The simulation reveals a 30°C thermal difference between collective and dispersed states at equilibrium, underscoring the critical role of swarming dynamics in energy localization. Compared to previous hyperthermia systems that primarily focused on targeted heat delivery, our strategy introduces dynamic reconfigurability to achieve tunable heating areas and scale‐invariant thermal stability. This capability effectively overcomes the severe heat dissipation typically encountered in varying physiological environments, as demonstrated in an in vivo rat bladder model, offering a more adaptable and robust platform for personalized photothermal therapy. This mechanism not only reduces systemic toxicity risks associated with high‐dose administration but also overcomes heat dissipation challenges inherent to traditional photothermal therapy.

Magnetically guided navigation achieved 91.7% delivery efficiency in tortuous geometries, and real‐time US imaging confirmed swarm integrity in dynamic flows (1.08–9.77 cm/s), revealing robust magnetic actuation and stable navigation of the microswarm in vascular environments. NIR‐triggered drug release established a synergistic chemo‐photothermal platform, achieving a 7‐fold improvement in cancer cell lethality compared to the dispersion‐based system. The microswarm's dual capacity for on‐demand photothermal modulation and precise drug delivery positions it as a promising platform for treating diseases in vascular and luminal/cavity environments, such as tumors and bacterial infections in hard‐to‐reach regions. By transcending the trade‐off between photothermal conversion efficiency and biosafety associated with high dosage administration, this work established an alternative design principle for photothermal agents without relying on sophisticated structural fabrication or modification. Future efforts will focus on in vivo validation and swarm optimization to enable collective intelligence for personalized medicine.

## 4 Experimental Section

### 4.1 Materials

Tris(hydroxymethyl)aminomethane (Tris, 99.9%), iron (III) chloride hexahydrate (FeCl_3_·6H_2_O, 99%), doxorubicin hydrochloride (DOX, 98%), ethylene glycol (EG, 99%), sodium acetate (NaAc, 99%), dopamine hydrochloride (98%), and polyethylene glycol (PEG, Mw = 20000) were purchased from Aladdin Chemicals. Hydrochloric acid (HCl, 37%) was purchased from Duksan (Korea). All the chemicals were used without further purification.

### 4.2 Preparation of Fe_3_O_4_ Nanoparticles

The Fe_3_O_4_ nanoparticles were prepared by a previously reported solvothermal method. In a typical procedure, FeCl_3_·6H_2_O (2.7 g) was initially dissolved in 80 mL of EG via continuous magnetic stirring, to form an orange solution, followed by the addition of NaAc (7.2 g) and PEG (2 g). After vigorous stirring for 12 h, the mixture was then sealed in the autoclave and heated to 200°C for 10 h, after which it was allowed to cool down to ambient temperature. The resulting black nanoparticles were collected using a permanent magnet and rinsed three times with deionized (DI) water to eliminate any remaining solvent. In the final step, the nanoparticles were dispersed in DI water for further use.

### 4.3 Preparation of Fe_3_O_4_@PDA Nanoparticles

A layer of PDA was modified on the Fe_3_O_4_ surface via a polymerization method. Initially, Tris (0.24 g) was dissolved in DI water (200 mL) with continuous sonication. The pH of the solution was adjusted to 8.5 using 1 m HCl, resulting in an alkaline buffer solution. This buffer was then transferred into a 500 mL round‐bottom flask, and Fe_3_O_4_ nanoparticles (200 mg) were added. The mixture was mechanically stirred and sonicated for 15 min, then 0.02 g of dopamine hydrochloride was added to the mixture. The polymerization reaction continued for 5 h with stirring. Finally, the product was collected using a permanent magnet and rinsed with DI water three times.

### 4.4 Drug Loading and Release

Fe_3_O_4_@PDA nanoparticles (1 mg) were suspended in *Tris* buffer (2 mL, pH = 8.0). DOX (2 mg) was then added to the Tris buffer with continuous stirring for 24 h. After that, Fe_3_O_4_@PDA‐DOX nanoparticles were collected and washed with DI water three times. The UV–vis spectra was obtained using the 5X‐diluted solution before and after loading. The loading weight of DOX was calculated as follows: *W*_Loading_ = *W*_Initial DOX_ − *W*_DOX in supernatant_. The amount of DOX was obtained with the calibration curve at the wavelength of 480 nm.

For drug release, the Fe_3_O_4_@PDA‐DOX nanoparticles (1 mg) were suspended in the buffer solution (2 mL, pH = 6.0) with continuous moderate shaking. At different time intervals, the released DOX was determined by measuring the UV–vis spectrum. Then, the solution was replaced with a fresh buffer solution, and the procedure was repeated several times. The 808 NIR laser‐triggered DOX release was investigated with laser (5.31 W/cm^2^) irradiation for 10 min at predetermined time intervals.

### 4.5 Controllable Photothermal Effect of Microswarm

To form the microswarm in the tank, a sphere magnet (D = 25 mm) was employed to generate the rotating magnetic field. Without further specification, the experimental parameters are set as follows: nanoparticle dose: 40 µg, magnetic field strength: 34.2 mT, frequency: 3 Hz, laser power density: 7.8 W/cm^2^. To obtain various nanoparticle densities of the microswarm, a conical magnetic field was applied to adjust the diameter of the microswarm, resulting in various nanoparticle densities. A NIR laser (808 °nm) was used to investigate the photothermal effect of the microswarm by measuring the temperature change. The laser was continuously irradiated on the microswarm until reaching the temperature equilibrium state, then the value of temperature was recorded and analyzed.

### 4.6 Simulations

The simulation results were obtained via COMSOL. The NIR (808 nm) with power densities of 1 and 3 W/cm^2^ was applied to the simulated particles (diameter of 300 nm). The number of particles was 361, and the simulated particles were preset as a collective and dispersive state, respectively, via regulating the particle distribution area. The temperature in the plot refers to the average temperature in the particle region.

### 4.7 Calculation of Photothermal Conversion Efficiency (PCE)

The photothermal conversion efficiency (PCE, η) of the nanoparticles in different states was calculated according to the standard Roper method [41]. Samples including the Fe_3_O_4_@PDA dispersion, Fe_3_O_4_@PDA swarm, bare Fe_3_O_4_ swarm, and pure water (as a background control) were prepared in containers with the same volume (0.3 mL). The samples were continuously irradiated by an 808 nm NIR laser. The real‐time temperature changes were monitored and recorded using an infrared thermal camera. The laser irradiation was maintained until the system reached a steady‐state maximum temperature (*T_max_*). Subsequently, the laser was turned off, and the natural cooling process of the system was continuously recorded until the temperature returned to the ambient surrounding temperature (*T_surr_*), thereby obtaining the complete heating and cooling curves.

Based on the obtained cooling curves, a dimensionless driving force temperature (θ) was introduced and calculated using the following equation: $\theta =\frac{T-T_{surr}}{T_{max}-T_{surr}}\;$, where *T* is the real‐time temperature during the cooling period. The cooling time (*t*) and the dimensionless temperature (θ) follow the relationship: $t=-\tau_{s}\ln\left(\theta \right)$. By plotting the cooling time (*t*) against −ln(θ), a linear fitting was performed. The system time constant for heat transfer (τ_*s*_) was determined as the reciprocal of the slope of this fitted line.

The overall heat transfer coefficient (*hS*) of the system was calculated using the obtained time constant (τ_*s*_): $hS=\frac{mC_{p}}{\tau_{s}}\;$, where *m* and *C_p_* are the mass and the specific heat capacity of the solvent (water, *C_p_* ≈ 4.2 J/(g · ^°^C)), respectively.

Finally, the photothermal conversion efficiency (η) was calculated using the energy balance equation: $\eta =\frac{hS\left(T_{max}-T_{surr}\right)-Q_{s}}{I(1-10^{-A_{808}})}\;,$where *Q_s_* represents the baseline thermal energy inputted by the solvent and the container, which was independently measured using pure water under laser irradiation conditions (*Q_s_* =  *hS* · Δ*T_water_*). *I* is the incident laser power (1.5 W), and *A*_808_ is the absorbance of the nanoparticles at the wavelength of 808 nm.

### 4.8 Controllable Locomotion of the Microswarm

The maze with tortuous channels was made by laser cutting of the acrylic sheet. A sphere magnet was mounted on a movable platform for magnetically controlled navigation of the microswarm. 40 µg of nanoparticles were added to the circular channel, then a rotating magnetic field was applied (magnetic field strength: 62.7 mT, input frequency: 3 Hz). Continuous navigation of the microswarm was performed in the maze with a velocity of 30 µm/s. To further verify the mobility of the microswarm in the dynamic environment, US imaging was used to track the microswarm in the vessel. A silicone tube with an inner diameter of 1.5 mm was used as the artificial vessel to mimic the human vascular environment. To create a flowing blood environment, a peristaltic pump was connected to the tube, continuously pumping the whole pig blood. The pulsatile mean blood flow velocity can be adjusted by the rotating speed of the peristaltic pump. Then, 100 µg of nanoparticles were injected into the tube to form the microswarm with the rotating magnetic field (magnetic field strength: 62.7 mT, input frequency: 3 Hz), and US imaging was used to track the locomotion process in the tube with a mean blood flow velocity of 6 cm/s. After that, the access rate of the microswarm in the bloodstream with various mean blood flow velocities (1.08, 2.17, 3.25, 4.34, 5.43, 6.52, 7.60, 8.68, 9.77 cm/s) was investigated. The access rate was calculated after upstream or downstream locomotion for a distance of 5 cm: Access rate = (Recycled weight)/(Initial weight) x 100%, where recycled weight refers to the weight of recycled nanoparticles from the tube after locomotion, and initial weight refers to the weight of injected nanoparticles into the tube.

### 4.9 In Vitro Cancer Cell Killing Test

The 4T1 cell line was propagated in RPMI‐1640 medium supplemented with 10% fetal bovine serum (FBS) and 1% penicillin/streptomycin. The cells were maintained in a humidified incubator at 37°C with 5% CO_2_. For the assays, 4T1 cells were seeded into 96‐well plates at 5 × 10^3^ cells/well and allowed to adhere for 24 h. Then the medium was exchanged with varying groups, including a blank control group, Fe_3_O_4_@PDA group, Fe_3_O_4_@PDA‐DOX + laser group, swarm + laser group, and swarm‐DOX + laser group. Cell viability was assessed in two settings: without light exposure (dark toxicity) after 24 h, and following a prescribed laser exposure (wavelength: 808 nm, power density: 5.31 W/cm^2^, duration: 10 min) after a 4‐h incubation with the samples, then a further 6 h of incubation post‐irradiation. Cell viability was quantified using a CCK‐8 assay, with the absorbance measured at 450 nm against a 650 nm reference wavelength on a Tecan Infinite 200 PRO plate reader. Finally, live/dead staining was conducted using Calcein‐AM/PI, with fluorescent images captured using a Nikon microscope. All measures were repeated three times.

### 4.10 Apoptosis Analysis via Flow Cytometry

To quantitatively evaluate the cell death pathways induced by the synergistic chemo‐photothermal therapy, an Annexin V‐FITC/PI dual‐staining assay was performed. Briefly, 4T1 cells at the logarithmic growth phase were seeded into 6‐well plates and incubated for 24 h to allow complete adhesion. The medium was then replaced with fresh medium containing either Fe_3_O_4_@PDA or Fe_3_O_4_@PDA‐DOX nanoparticles. To induce microswarm formation, a rotating magnetic field (62.7 mT, 3 Hz) was applied for 1 min, followed by a 4 h co‐incubation. For the laser‐treated groups, cells were exposed to an 808 nm NIR laser (5.31 W/cm^2^) for 10 min. After a further 15 h of incubation, both the culture supernatant (containing late apoptotic and necrotic cells) and the adherent cells were carefully collected. Adherent cells were detached using EDTA‐free trypsin to preserve membrane integrity and phosphatidylserine exposure required for Annexin V binding. The pooled cells were centrifuged (1000 rpm, 5 min, 4°C) and washed with ice‐cold PBS. Subsequently, the cell pellets were resuspended in Binding Buffer and stained with Annexin V‐FITC and Propidium Iodide (PI). The mixture was incubated for 15–20 min at room temperature in the dark. Finally, Binding Buffer was added to each tube, and the samples were immediately analyzed using a flow cytometer (Beckman Coulter, CytoFLEX). The acquired data were analyzed to quantify the proportions of viable (Annexin V^−^/PI^−^), early apoptotic (Annexin V^+^/PI^−^), late apoptotic (Annexin V^+^/PI^+^), and necrotic (Annexin V^−^/PI^+^) cells.

### 4.11 In Vivo Validation in the Rat Bladder Model

All animal experiments were conducted in male Sprague–Dawley (SD) rats. Before the experiment, the rats were anesthetized. For the intravesical administration, 50 µL of the Fe_3_O_4_@PDA‐DOX nanoparticle suspension (dose: 200 µg) was injected into the bladder cavity. To evaluate the in vivo swarming and navigation capabilities, a commercial ultrasound imaging system (EDAN, Acclarix AX3) was used to continuously monitor the bladder region in B‐mode. An external rotating magnetic field (magnetic field strength: ∼32 mT, input frequency: 4 Hz) was applied near the bladder. Under real‐time ultrasound guidance, the dynamic assembly of the dispersed nanoparticles into a dense microswarm was recorded. The controllable and reversible locomotion of the microswarm was achieved by dynamically manipulating the position and direction of the external magnetic field, steering the swarm along the irregular inner wall of the bladder to predetermined targeted positions and back to the initial position.

For the in vivo photothermal amplification evaluation, the tests were divided into two states: the dispersive state (without magnetic actuation) and the swarming state (with magnetic actuation). The targeted bladder region was continuously irradiated by an 808 nm NIR laser at a power density of ∼5.3 W/cm^2^. The real‐time temperature variations and infrared thermal images of the rats were recorded using an infrared thermal camera. After the experiments, the rats were euthanized according to standard ethical protocols.

### 4.12 Characterization and Measurements

TEM images were obtained using the JEOL Model JEM‐2011 System. Zeta potential and DLS tests were performed using a particle analyzer (ZS90, LC10‐HS). The XRD patterns was measured by an X‐ray diffractometer (Bruker D8 Advance). The magnetic hysteresis loops were measured by a vibrating sample magnetometer (VSM, LakeShore 7404/8604).

## Author Contributions

Q.L.W. performed conceptualization. Q.L.W., L.S., and J.J.W. performed the methodology. Q.L.W., B.W., L.S., Y.H.J., Z.X.Y., Q.Q.W., J.J.W., and L.Z. performed the investigation. Q.L.W. performed visualization. B.W., Q.Q.W., J.J.W., and L.Z. performed supervision. Q.L.W. wrote the original draft. Q.L.W., L.S., B.W., Q.Q.W., J.J.W. and L.Z. performed writing – review & editing.

## Conflicts of Interest

The authors declare no conflicts of interest.

## Supporting information




**Supporting File 1**: advs76128‐sup‐0001‐SuppMat.docx.

**Supporting File 2**: advs76128‐sup‐0002‐VideoS1‐S6.zip.

## Data Availability

The data that support the findings of this study are available from the corresponding author upon reasonable request.
