# Supplementary material for: Photothermal Amplification via Nanorobotic Swarming Dynamics
Source: Adv Sci (Weinh). 2026 Jun 15:e76128. Online ahead of print. doi: 10.1002/advs.76128 (PMC13337063; doi:10.1002/advs.76128)
Supplement: Supplementary file 1 — Supporting File 1: advs76128‐sup‐0001‐SuppMat.docx. [file ADVS-9999-e76128-s001.docx]

Photothermal Amplification via Nanorobotic Swarming Dynamics

Qinglong Wang^1,2#*^, Lin Su^3#^, Zhengxin Yang^4#^, Yihang Jiang^2^, Haojin Yang^2^, Qianqian Wang^5,6*^, Li Zhang^2,7,8^, Ben Wang^9*^, Jiajia Wang^1*^

^1^School of Public Health, Guangzhou Medical University, Guangzhou 511436, China.

^2^Department of Mechanical and Automation Engineering, The Chinese University of Hong Kong, Hong Kong SAR, China.

^3^Department of Industrial and Systems Engineering, The Hong Kong Polytechnic University, Hong Kong, China.

^4^The Suzhou Institute of Biomedical Engineering and Technology, Chinese Academy of Sciences, Suzhou 215000, China.

^5^Jiangsu Key Laboratory for Design and Manufacturing of Precision Medicine Equipment, School of Mechanical Engineering, Southeast University, Nanjing 211189, China.

^6^Department of Robotics, School of Mechanical Engineering, Southeast University, Nanjing 211189, China.

^7^Chow Yuk Ho Technology Centre for Innovative Medicine, The Chinese University of Hong Kong, Hong Kong SAR, China.

^8^Department of Surgery, The Chinese University of Hong Kong, Hong Kong SAR, China.

^9^College of Chemistry and Environmental Engineering, Shenzhen University, Shenzhen 518000, China.

#These authors contribute equally to this work.

*Corresponding author. email: qinglongwang@link.cuhk.edu.hk (Q.L.W.); benwang@szu.edu.cn (B.W.); qqwang@seu.edu.cn (Q.Q.W.); wangjj@gzhmu.edu.cn (J.J.W.).

**Video S1:** Temperature change of samples in various states.

**Video S2:** Microswarm formation and reconfiguration.

**Video S3:** The simulation of temperature change in collective and dispersive states.

**Video S4:** Controllable locomotion of the microswarm in the channel.

**Video S5:** US imaging-guided tracking of the microswarm.

**Video S6:** *In vivo* US imaging-guided navigation and photothermal amplification of the microswarm in bladder.

**Figure S1.** Zeta potential of Fe_3_O_4_ and Fe_3_O_4_@PDA nanoparticles in DI water with pre-ultrasound processing for 5 min.

**Figure S2.** Areal density of microswarm with various diameters, the insert amplified figure shows the areal density of dispersion.

**Figure S3.** The photo of Fe_3_O_4_@PDA-DOX dispersion, and the inset pictures show the final temperature.

**Figure S4.** The influence of the distance between the NIR and the swarm on the photothermal effect.

**Figure S5.** Standard calculations of the photothermal conversion efficiency (PCE).

**Figure S6.** A standard curve of Dox concentrations in buffer solution.

**Figure S7.** UV-vis absorbance spectra of DOX released from Fe_3_O_4_@PDA-DOX nanoparticles.

**Figure S8:** Flow cytometry analysis of cancer cell apoptosis induced by different treatments using Annexin V-FITC/PI dual staining.

**
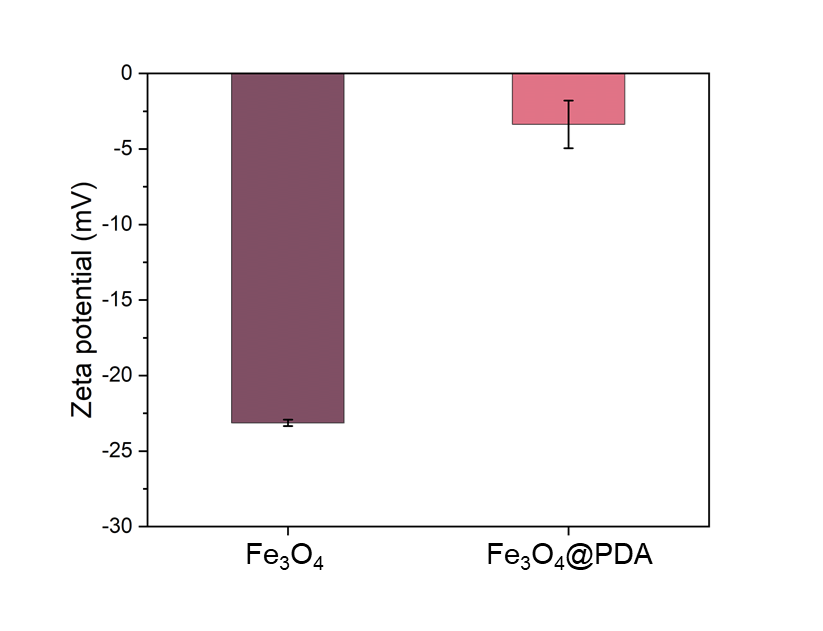
Figure S1.** Zeta potential of Fe_3_O_4_ and Fe_3_O_4_@PDA nanoparticles in DI water with pre-ultrasound processing for 5 min.

**
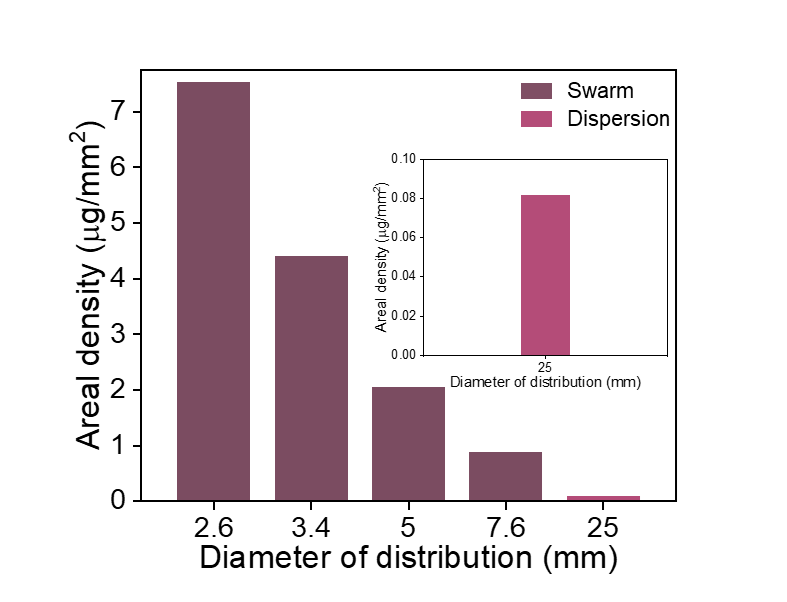
Figure S2.** Areal density of microswarm with various diameters, the insert amplified figure shows the areal density of dispersion.

**
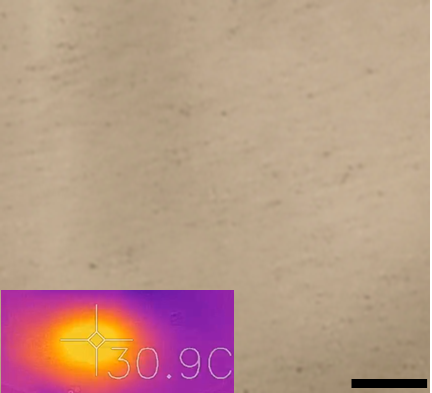
Figure S3.** The photo of Fe_3_O_4_@PDA-DOX dispersion, and the inset pictures show the final temperature. Scale bar: 2 mm.

**
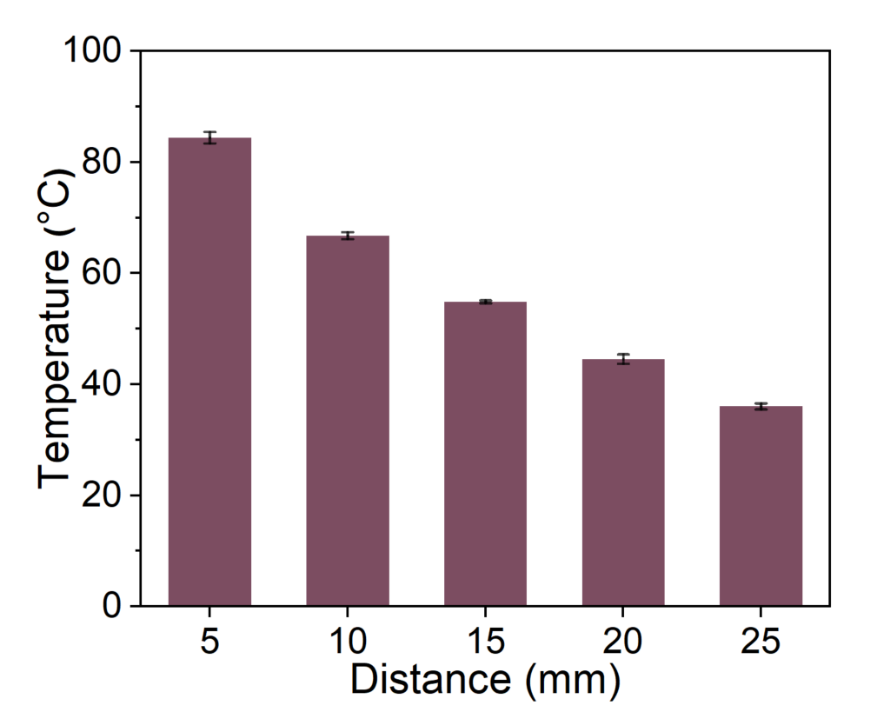
Figure S4.** The influence of the distance between the NIR and the swarm on the photothermal effect. Magnetic field strength: 37.2 mT, input frequency: 3 Hz, tank diameter: 2.5 mm.

**
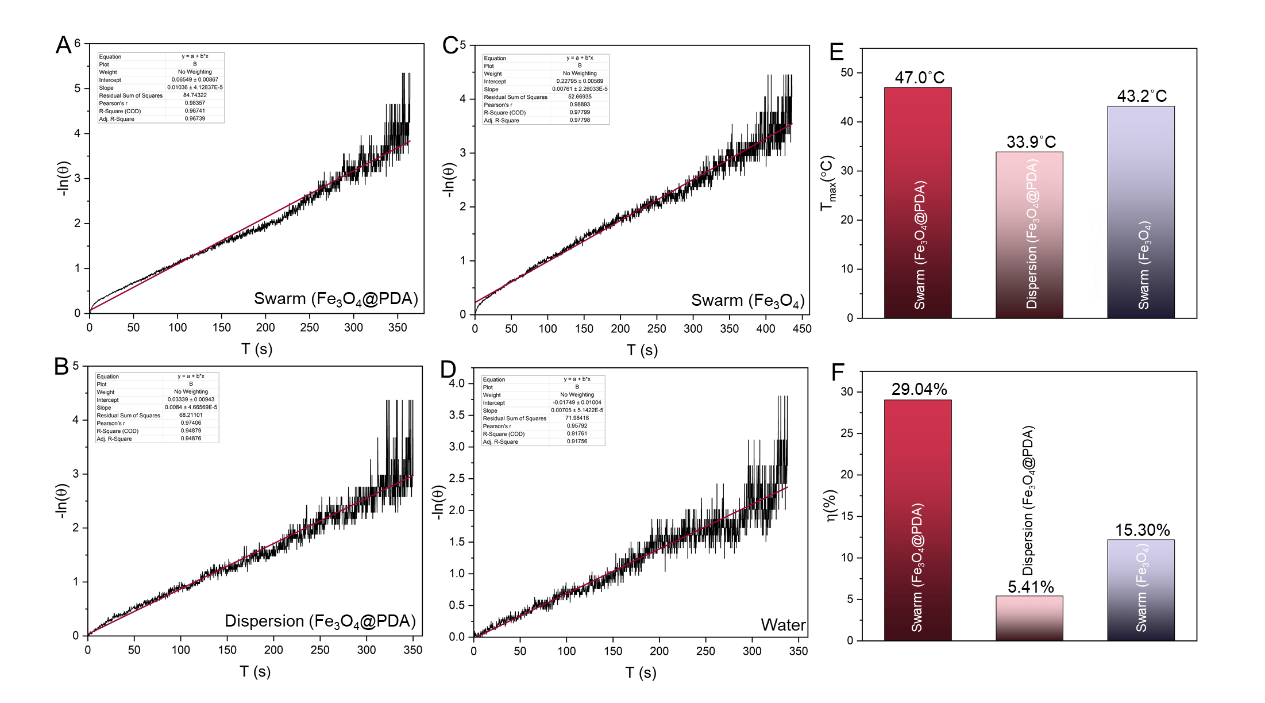
Figure S5.** Standard calculations of the photothermal conversion efficiency (PCE). Linear time data versus $-\ln(\theta)$obtained from the cooling period of (A) Swarm (Fe_3_O_4_@PDA), (B) Dispersion (Fe_3_O_4_@PDA), (C) Swarm (Fe_3_O_4_), and (D) pure water. (E) The maximum steady-state temperatures ($T_{max}$) achieved by different groups under 808 nm laser irradiation. (F) The calculated apparent photothermal conversion efficiencies ($\eta$) of the corresponding groups.

**
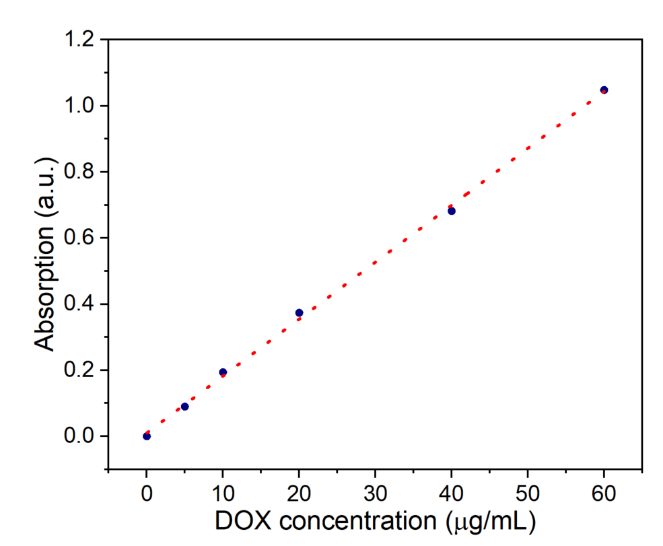
Figure S6.** A standard curve of Dox concentrations in buffer solution.


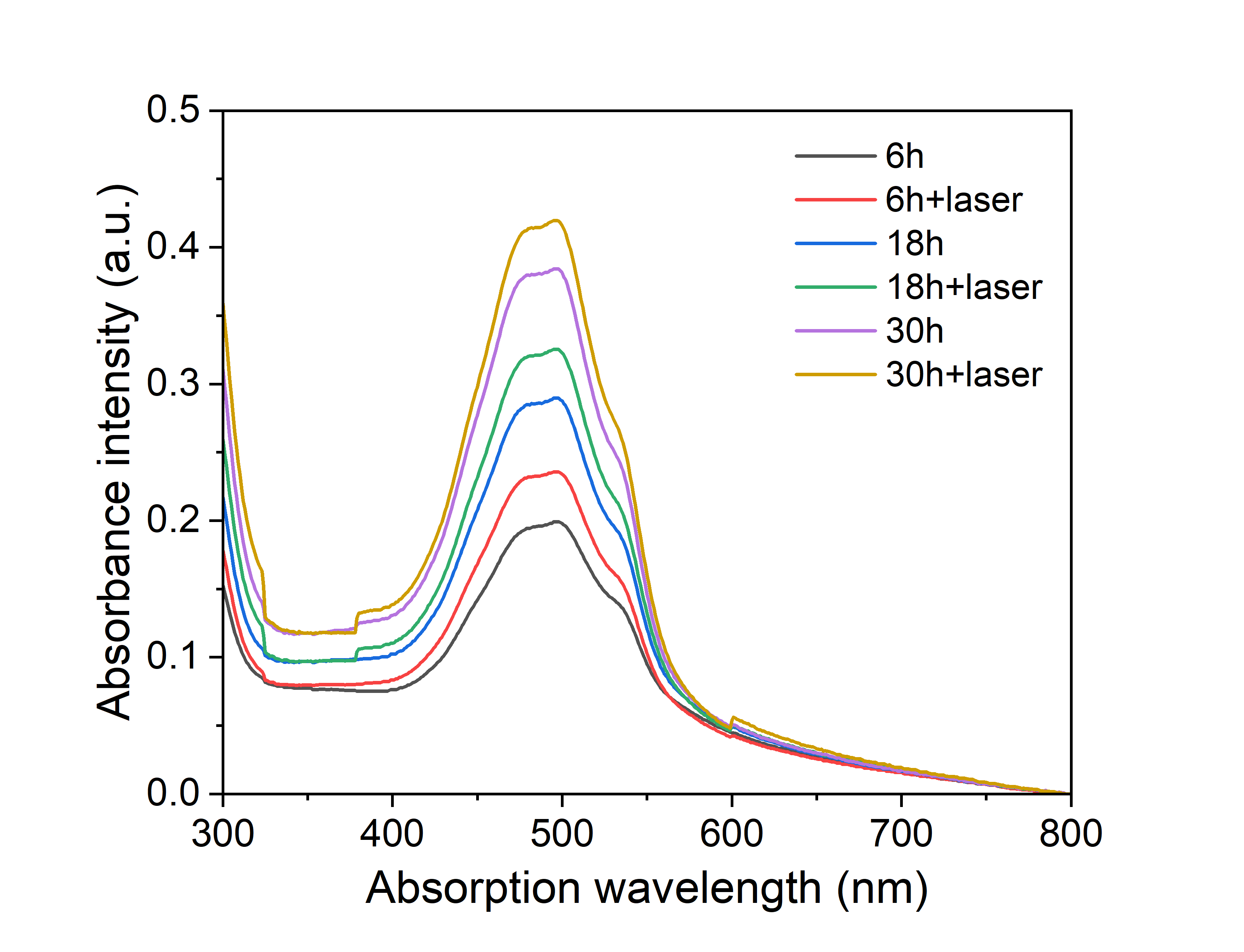
**Figure S7.** UV-vis absorbance spectra of DOX released from Fe_3_O_4_@PDA-DOX nanoparticles (1 mg) over time, and the samples were treated with laser irradiation at different time points.


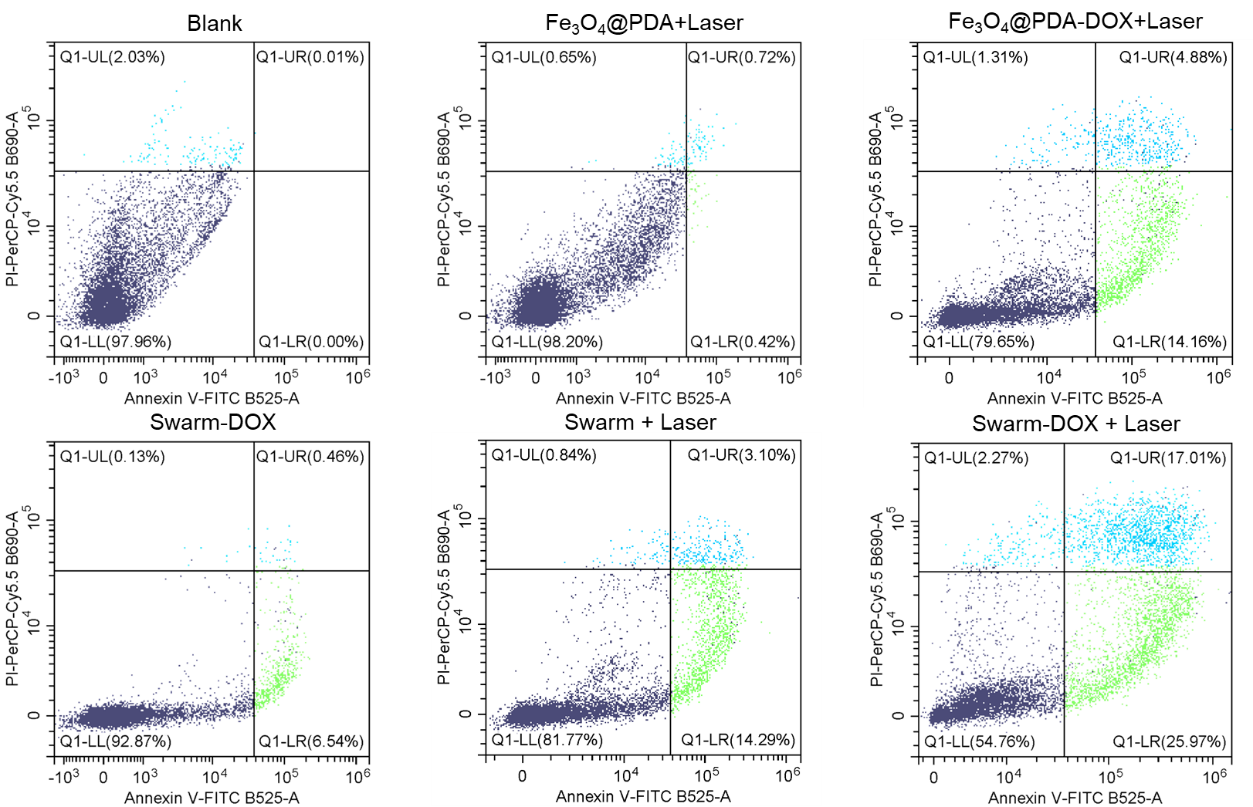
**Figure S8.** Flow cytometry analysis of cancer cell apoptosis induced by different treatments using Annexin V-FITC/PI dual staining.
